# Dose Comparison of Dexmedetomidine Sedation following Spinal Anesthesia: Parturient versus Nonpregnant Women—A Randomized Trial

**DOI:** 10.1155/2020/1059807

**Published:** 2020-07-27

**Authors:** Ming Xiong, Biyun Chen, Zurong Hu, Somdatta Gupta, Zhitao Li, Jiping Liu, Jing He, Shivani Patel, Jean Daniel Eloy, Bo Xu

**Affiliations:** ^1^Department of Anesthesiology, General Hospital of Southern Theatre Command of PLA, Guangzhou, China; ^2^Department of Anesthesiology and Preoperative Medicine, Rutgers-NJMS, Newark, NJ 07101, USA; ^3^Department of Anesthesiology, Foshan Maternal and Child Health Care Hospital, Foshan, China; ^4^Department of Anesthesiology, Guangdong Province Hospital for Women and Children Health Care, Guangzhou, China; ^5^Department of Anesthesiology, Peking University Shenzhen Hospital, Shenzhen, China

## Abstract

**Background:**

This study was designed to investigate and compare the effective doses of dexmedetomidine for sedation in parturient patients who underwent Cesarean section (CS) and nonpregnant women who underwent elective gynecologic surgery.

**Methods:**

The study comprised 60 females aged between 25 and 35. They were divided into two groups. The parturient group received a bolus dose of dexmedetomidine over 15 min after the delivery of the fetus and placenta. In the nonpregnant women group, a bolus of dexmedetomidine was administered intravenously upon the completion of spinal anesthesia. The subsequent dose required by patients in each group was then determined through a modified two-stage Dixon up-and-down sequential method. Probit analysis was used to calculate the ED50 and the ED95 of dexmedetomidine for adequate sedation.

**Results:**

The ED50 of dexmedetomidine for adequate sedation in parturient patients was 1.58 *μ*g/kg (1.51–1.66 *μ*g/kg); in nonpregnant women, it was 0.96 *μ*g/kg (0.91–1.01 *μ*g/kg) (95% CI). The ED95 of dexmedetomidine in parturients was 1.80 *μ*g/kg (1.70–2.16) *μ*g/kg and that of nonpregnant women was 1.10 *μ*g/kg (1.04–1.30 *μ*g/kg) (95% CI). The ED50 in parturients was significantly higher than that in nonpregnant women (*P* < 0.05).

**Conclusion:**

The ED50 of dexmedetomidine for target sedation in parturients who received spinal anesthesia for CS is greater than 1.5 times that in nonpregnant women who received spinal anesthesia for lower abdominal gynecologic surgery. This study postulates that the dose of dexmedetomidine required to achieve optimal sedation following spinal anesthesia is much higher in parturients than in nonpregnant women undergoing gynecologic surgeries. This trial is registered with NCT02111421.

## 1. Introduction

Spinal anesthesia is widely employed in Cesarean section (CS), due to its advantages of spontaneous ventilation, muscle relaxation, and rapid onset. However, patients may experience fear and anxiety while remaining awake during surgery. Physical exhaustion combined with emotional stress is seen in many parturients, especially in those who have endured a trial of labor preoperatively. Even after delivery, parturients continue to experience emotional upheavals and a sympathetic surge with hemodynamic changes. All of these responses can be significantly attenuated by administering a sedative or hypnotic drug after spinal anesthesia during CS, which would also enable the patient to get some rest after childbirth.

Oxytocin, often given for uterine atony or excessive bleeding to enhance uterine contractility in CS [1], can commonly cause nausea, vomiting, and chest pain—symptoms that may already be present due to peritoneal traction and delivery. These side effects, too, can be mitigated by a sedative/hypnotic drug that can be given to supplement the spinal anesthesia during CS immediately after delivery.

Dexmedetomidine is a highly selective *α*2-adrenergic receptor agonist that provides sedative, analgesic, and anxiolytic properties with minimal respiratory depression [2]. There are several reports of dexmedetomidine being used for amnesia in CS under general anesthesia [3–5]; however, very few studies have actually demonstrated its use in CS under spinal anesthesia. Thus, the objective of this study was to ascertain the effective dose of dexmedetomidine required for sedation immediately after delivery of the fetus in CS, as well as to compare the difference in optimum doses in this population versus in nonpregnant women.

## 2. Methods

This study was approved by the local ethics board (General Hospital of Southern Theatre Command of PLA Medical Ethics Committee) on February 3rd, 2015, and written informed consent was obtained from all subjects participating in the trial. The trial was registered prior to patient enrollment at Clinicaltrials.gov (NCT 02111421, Principal investigator: Bo Xu, Date of registration: April 7, 2014).

The study comprised 60 females of ages 25–35 and ASA statuses I-II. They were divided into two categories: (i) 31 parturients between 35 and 42 weeks' gestation scheduled for elective CS under spinal anesthesia and (ii) 29 nonpregnant women scheduled for elective gynecologic surgery under spinal anesthesia. Exclusion criteria included pregnancy-induced hypertension; a history of neurological deficits, coagulation disorders, clinically significant cardiac, respiratory, metabolic, kidney, and liver dysfunction (albumin <35 g/L); allergy to the planned medications; diabetes; morbid obesity (body mass index > 35 kg/m2); or contraindications to spinal anesthesia. Subjects who experienced sensory block above T4 level or those who had hypotension (mean arterial pressure <50 mmHg) due to spinal anesthesia were excluded, as were those in whom the intraoperative blood loss exceeded 15 mL/kg.

All patients were required to fast for 8 hours before surgery and they received no premedication. Upon arrival into the operating room, an intravenous cannula was inserted into the dorsum of the hand through which a bolus of 8–10 mL/kg crystalloid was infused before administering spinal anesthesia. Electrocardiogram, pulse oximetry (SpO_2_), heart rate (HR), noninvasive blood pressure (BP), and Narcotrend (Monitor Techink Company, Germany) were monitored at 5 min intervals continuously. Nonpregnant women were placed in the lateral decubitus position, and parturients were placed in the right lateral decubitus position. Each of the 2 groups had a lumbar puncture at the level of L2-3 or L3-4. These levels were determined based on superficial anatomical landmarks alone and therefore have been given in a range. Clear cerebrospinal fluid was aspirated, and 7–10 mg of 0.5% bupivacaine (mixture of 0.75% bupivacaine and 10% glucose solution) was injected into the subarachnoid space. After delivering the spinal anesthetic, an epidural catheter was inserted 3–4 cm cephalad and secured in place, to be used with 2% lidocaine only if spinal anesthesia proved to be inadequate. (In each case, the epidural catheter was removed immediately after the surgery.)

Patients in both groups were then positioned supine, and the operating table in the parturient group was tilted at 15° on the left lateral side to minimize aortocaval compression. They received supplemental oxygen at 3 L/min via face mask for the duration of the procedure.

Sensory block was assessed at each dermatomal level bilaterally for loss to pinprick, cold, and light touch sensation, to ensure that the level of anesthesia reached up T6–T8. A level of T6 was used for parturients while T8 was primarily used for nonpregnant patients.

In the parturient group, after the delivery of fetus and placenta, all patients received intravenous infusion of dexmedetomidine (Jiangsu Hengrui Medicine Co. Ltd., Lianyungang, China) at a concentration of 4 *μ*g/mL over 15 min through an Injectomat TIVA Agilia syringe pump (Fresenius Kabi Laboratories, France). In the nonpregnant women group, after ensuring an adequate level of sensory block from spinal anesthesia, the same concentration of 4 *μ*g/mL dexmedetomidine was administered over 15 min. No other sedative or analgesic was administered prior to the dexmedetomidine infusion.

Dosing of dexmedetomidine was based on the patient's preoperative body weight. However, considering the physiological changes of pregnancy, the body weight of the parturient was calculated as follows: effective body weight = preoperative weight − [placental weight + neonatal weight + amniotic fluid]. The dose for each group was determined by a modified two-stage Dixon up-and-down sequential method [6, 7] where the dose was increased following a nonresponse (inadequate sedation) and decreased following a response (effective sedation).

In stage I of the Dixon up-and-down sequential method, the initial dose designated for each group was 1.0 *μ*g/kg. If the desired degree of sedation was not achieved for a particular patient, we increased the dose for the next patient by 0.1 *μ*g/kg. If sedation was adequate, the dose was decreased by the same amount of 0.1 *μ*g/kg. The process was repeated until three crossover points were identified. Subsequently, we commenced stage II where the last administered dose in stage I was assigned as the initial dose of stage II. The up-and-down sequence was reimplemented after decreasing the step-up/down size to 0.05 *μ*g/kg. This stage was terminated once another set of four crossover points were determined. Hence, the total number of patients required for the study could not be calculated beforehand.

Each patient's sedation level was assessed every 5 minutes after the dexmedetomidine infusion was started, for a total duration of 30 minutes. We used the observer's assessment of alertness/sedation (OAA/S) scale (Table 1); adequate sedation was defined as an OAA/S score of ≤3 at any point in time during the 30 min [8]. HR, MAP, OAA/S scale, and Narcotrend index were recorded at baseline T0 (prior to dexmedetomidine administration) as well as after 5 min (T1), 10 min (T2), 15 min (T3), 20 min (T4), 25 min (T5), and 30 min (T6). The Narcotrend index and OAA/S scores were independently recorded by two different observers. Each observer was blinded to the dexmedetomidine dose administered to the patients. One observer recorded the Narcotrend index score, following which a different observer recorded the OAA/S, unaware of the previous assessment.

Adverse events (e.g., nausea, vomiting. or shivering) were recorded. Bradycardia (HR < 50) was treated with 0.5 mg IV atropine. Intraoperative hypotension (MAP < 50 mmHg or SBP < 80 mmHg) with bradycardia was managed by increasing the infusion rate of Ringer's lactate and concurrently administering 5 mg IV ephedrine. Resuscitation measures were reassessed every 1 min so that boluses could be repeated as necessary.

Once the dexmedetomidine infusion was complete, precautions were taken to keep the airway patent by avoiding airway obstruction provoked by episodic jaw relaxation. Respiratory rate <10/min or SpO_2_ < 92% was managed with manual ventilation. Upon completion of the CS, parturients were observed to the postanesthesia care unit for at least 1 hour.

### 2.1. Statistical Analyses

Statistical analyses were performed using SPSS statistical package version 13.0 for Microsoft Windows. Data was expressed as the mean ± standard deviation or number of patients. For numerical data, the Kolmogorov–Smirnov test was used for a normal distribution, followed by Student's *t*-test for normally distributed data, and the Mann–Whitney *U* test for nonnormally distributed data between the groups. Changes in HR and MAP were analyzed using a two-way analysis of variance for repeated measures within the groups. The up-and-down data was analyzed by Probit analysis to derive the ED50 (median effective dose) and ED95 (dose required for desired effect in 95% of the population exposed to it) with 95% CI and plot the dose-response curves. Student's *t*-test with Bonferroni correction was used to evaluate ED50 differences between the two groups. The correlation between the Narcotrend index and OAA/S scale was analyzed using Spearman's rank correlation. *P* < 0.05 was considered statistically significant.

## 3. Results

A total of 33 parturients and 29 nonpregnant women were enrolled for the study. One parturient whose MAP dropped to 46 mmHg was excluded, as was another who had to be kept awake and alert to cooperate with the surgeons throughout the operation. Therefore, a total of 60 patients (31 parturients and 29 nonpregnant women) were included in the final analysis. The patients' demographic data are presented in Table 2.

**Figure 1:**
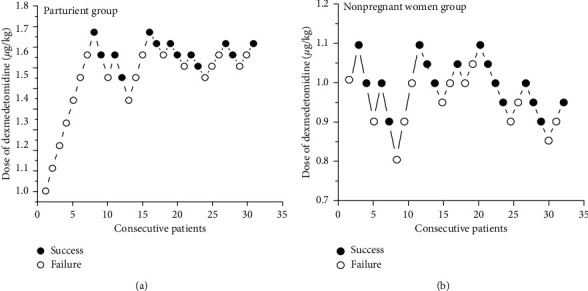
Responses of consecutive patients receiving dexmedetomidine sedation in postpartum parturient group and nonpregnant women group according to the modified up-and-down sequence.

In the parturient group, there were 13 successful cases and 18 failed cases; in the nonpregnant group, there were 16 successful cases and 13 failed cases (Figure 1).

**Figure 2:**
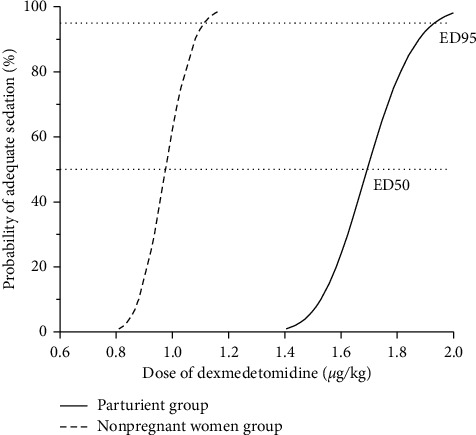
Dose-response curves plotted from the Probit analysis of individual dexmedetomidine sedation in postpartum parturient group and nonpregnant women group. ED50, the median effective dose; ED95, the 95% effective dose.

**Figure 3:**
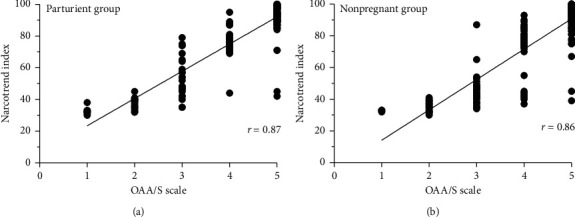
The correlation between Narcotrend index and OAA/S scale under dexmedetomidine sedation with spinal anesthesia in both groups. *r*, correlation coefficient.

By Probit analysis, the ED50 of dexmedetomidine for adequate sedation in postpartum parturients was 1.58 *μ*g/kg (1.51–1.66 *μ*g/kg); in nonpregnant women it was 0.96 *μ*g/kg (0.91–1.01 *μ*g/kg) (95% confidence interval (CI)). The ED95 of dexmedetomidine in postpartum patients was 1.80 *μ*g/kg (1.70–2.16) *μ*g/kg and that of nonpregnant women was 1.10 *μ*g/kg (1.04–1.30 *μ*g/kg) (95% CI). The ED50 in postpartum parturients was significantly higher than that in nonpregnant women (*P* < 0.05). Dose-response curves plotted from the Probit analysis are presented in Figure 2. The correlation coefficients between the Narcotrend index and OAA/S scale for postpartum parturients and nonpregnant women were 0.87 and 0.86, respectively (*P* < 0.05) (Figure 3).

**Figure 4:**
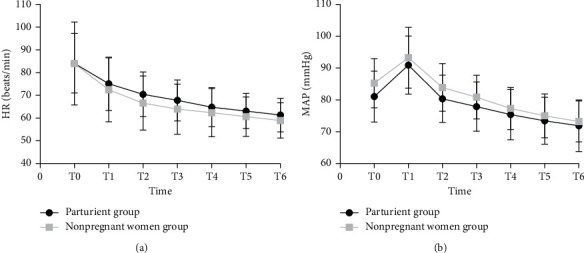
The HR (a) and MAP (b) in both groups at different time points. HR, heart rate; MAP, mean arterial pressure; T0, before dexmedetomidine administration; T1, T2, T3, T4, T5, and T6, 5 min, 10 min, 15 min, 20 min, 25 min, and 30 min after dexmedetomidine infusion started.

Compared with T0, a biphasic change in MAP in both groups was observed, characterized by a significant increase at T1 followed by a decrease at T3 (Figure 4(a)). In addition, HR in both groups started to decline at T1 from the baseline at T0 (Figure 4(b)). Prior to the administration of dexmedetomidine, 3 parturients complained of abdominal pain; in one patient the abdominal pain was additionally accompanied by vomiting and chest pain. However, all of these symptoms resolved once intravenous dexmedetomidine infusion was started. None of the patients required treatment with atropine or ephedrine during surgery. No patient required local anesthetics via the epidural catheter, and no shivering events occurred. All patients maintained a SpO_2_ above 96% during the surgery.

## 4. Discussion

Our study found that the ED50 of dexmedetomidine for effective sedation in postpartum parturients was 1.58 *μ*g/kg (1.51–1.66 *μ*g/kg) and in nonpregnant women it was 0.96 *μ*g/kg (0.91–1.01 *μ*g/kg) (95% CI). These results indicate that the ED50 of dexmedetomidine for adequate sedation in postpartum parturients is greater than 1.5 times that in nonpregnant women, all of whom had received spinal anesthesia for their respective surgeries. Therefore, a higher dose of dexmedetomidine should be used to achieve target sedation goals for parturients after childbirth in CS under spinal anesthesia.

Current literature has proposed that the changes in progesterone and circulating endorphins during pregnancy might reduce the effective minimum alveolar concentration (MAC) for most inhalation agents [9–11]. Similarly, Gin reported that, for pregnant women at 7–13 weeks' gestation, the dose of thiopental for hypnosis was 17% lower and for anesthesia was 18% lower than for nonpregnant women [12]. Similarly, Mongardon suggested that the propofol dose and predicted propofol effect-site concentration for loss of consciousness are decreased during early pregnancy [13]. Similar to early pregnancy, MAC values for isoflurane continued to be decreased 24–36 hours after delivery and gradually returned to normal by 72 hours postpartum [14]. Thus, we initially started with a hypothesis that the dose of dexmedetomidine required to adequately sedate parturients may be less than that for nonpregnant women. However, contrary to our own hypothesis, the results we obtained served only to indicate that the dose of dexmedetomidine necessary to sedate parturients adequately is significantly higher than the dose required for nonpregnant women undergoing surgery under spinal anesthesia.

In a study similar to ours, Neuman used dexmedetomidine to facilitate awake fiber-optic intubation in a parturient with spinal muscular atrophy type III who was scheduled for CS under general anesthesia; he used a total dose of 1.5 *μ*g/kg bolus followed by an infusion of 1 *μ*g/kg/h [5]. This was significantly greater than the 0.92 *μ*g/kg bolus dose that was necessary for sedation during awake fiber-optic intubation in their institute previously.

Pharmacokinetic differences between parturients and nonpregnant women may account for the difference in the dose of dexmedetomidine required to achieve target sedation in the 2 groups. Pregnancy-induced increase in circulatory blood volume (by 30–45%), as well as an increase in cardiac output immediately postpartum, results in an increase in the apparent volume of distribution of a drug. By pharmacokinetic definition, a larger volume of distribution of a drug demands a larger dose to reach an identical concentration. This can explain why larger doses of dexmedetomidine are required in parturients than in nonpregnant women to achieve the same effect.

The correlation coefficients in both groups indicate a good correlation between Narcotrend index and OAA/S scale. However, even when the Narcotrend index score fell below 40 or even 30, more than half of the patients in successful cases were still arousable. This suggests that patients are quite easily arousable with dexmedetomidine sedation, unlike sedation from other agents, e.g., propofol, which causes much greater impairment.

The sedative-hypnotic effects of dexmedetomidine are mediated by the hyperpolarization of *α*2-adrenergic neurons in the locus cerulean of the brain stem, in contrast with sedation induced by *λ*-amino butyric acid agents [15]. Due to the difference in mechanisms of sedation in dexmedetomidine versus other agents, progesterone and circulating endorphins during pregnancy have different effects on them; therefore, to achieve the same target sedation, the dose of dexmedetomidine may need to be higher, versus other agents where the dose may need to be lower. Furthermore, visceral traction during surgery and side effects of uterotonic drugs also require higher doses of dexmedetomidine to attenuate these responses.

Yu et al. have shown that dexmedetomidine undergoes placental transfer with a fetal/maternal (*F*/*M*) ratio of 0.76 [15], indicating that dexmedetomidine can easily pass through the placental barrier. Nonetheless, it also exhibits significant placental tissue retention with relatively little amount transported to the fetal circulation due to its high lipophilicity, thus limiting its effects on the newborn [5]. However, being an *α*2-adrenoceptor agonist, dexmedetomidine has a direct effect on human myometrium and can enhance the frequency and amplitude of uterine contractility [16]. For this reason, we started the dexmedetomidine infusion only after the delivery of the fetus and placenta to avoid the above-stated adverse events. Moreover, dexmedetomidine has an onset of action of approximately 15 min [17]; administering dexmedetomidine after the delivery of the baby ensured unimpaired communication between the mother and her newborn in the immediate postpartum period. Hypotension and severe bradycardia (caused by rapid dexmedetomidine infusion) were avoided by slowly infusing a loading dose over 15 min. This different approach not only provided a stable hemodynamic state for the patient, but also served to maintain excellent sedation for the short procedure without prolonging the time of surgery.

In our study, we found that dexmedetomidine produces a biphasic change in MAP and a significant reduction in HR. It is surmised that sympatholysis from dexmedetomidine and the consequent increased vagal tone are involved in the slowing of the HR; the initial increase of MAP is thought to be due to vasoconstriction from the stimulation of peripheral postsynaptic *α*2B-adrenergic receptors; the subsequent decrease in MAP is due to presynaptic *α*2A-adrenergic receptor-stimulated sympatholysis [18]. However, due to the slow infusion of dexmedetomidine, none of our patients developed clinically significant hemodynamic disturbances that required intervention.

Our study has certain limitations: for the lack of a better clinical scoring system to assess sedation in the patient, we used the subjective and potentially disadvantageous OAA/S scale, which utilizes extrinsic stimulation such as calling, prodding, or shaking (unpleasant for the patient) to evaluate the neurological status. Furthermore, the results are dependent on the assessor and hence variable.

Another limitation is that high spinal anesthesia (up to T6) in CS can decrease afferent sensory transmission to produce sedative effects [19, 20]; it can modify the reticulothalamocortical response and consequently affect the observed results. We attempted to keep the level of sensory block no higher than T6 to minimize spinal anesthesia-induced sedation; however, it is possible that a sensory block level up to T6 could still have affected the results.

Since ED95 is beneficial for anesthesiologists under most clinical circumstances, we analyzed the up-and-down data by a Probit analysis to provide the ED50 and ED95 and their 95% CIs. A possible criticism of our study may be the accuracy of our ED95 and 95% CI calculations by Probit analysis. Probit analysis could provide all predicted values from ED1 to ED99 based on its algorithm; of these, the ED50 and ED95 and their 95% CIs values are presented in Results. The data we obtained using the up-and-down method was concentrated around the median points rather than the spread. Therefore, Probit analysis could provide an accurate ED50, but only predict the ED95 and their 95% CIs values; consideration is needed to use these values for clinical reference.

We also recognize that sedation during CS to supplement neuraxial anesthesia is not considered to be standard practice in many areas of the world. However, in a certain group of patients, anxiety may be a formidable factor. This, along with cultural differences in standard practice, may lead to the need for sedation. The purpose of this study was to determine the dose in those patients.

In summary, the ED50 of dexmedetomidine for target sedation in parturients who received spinal anesthesia for CS is greater than 1.5 times that in nonpregnant women who received spinal anesthesia for lower abdominal gynecologic surgery. This study indicates that the dose of dexmedetomidine required to achieve optimal sedation following spinal anesthesia is much higher in parturients than in nonpregnant women undergoing elective surgery.

## Figures and Tables

**Table 1:** Observer's assessment of alertness/sedation (OAA/S) scale.

Scale	Responsiveness	Speech	Facial expression	Eyes
5	Responds readily to name spoken in normal tone	Normal	Normal	Clear, no ptosis
4	Lethargic response to name spoken in normal tone	Mild slowing or thickening	Mild relaxation	Glazed or mild ptosis
3	Responds only after name is spoken loudly and/or repeatedly	Slurring or prominent slowing	Marked relaxation (slack jaw)	Glazed and marked ptosis
2	Responds only after mild prodding or shaking	Few recognized words		
1	Does not respond to mild prodding or shaking			

**Table 2:** Demographic data of the study subjects.

	Parturient group (*n* = 31)	Nonpregnant women group (*n* = 29)	*P* value
Age (years)	28.70 ± 3.46	29.17 ± 5.27	0.69
Height (cm)	160.64 ± 5.63	160.97 ± 6.12	0.83
Weight (kg)	69.23 ± 8.56	54.95 ± 8.04	<0.001
Fetal weight (kg)	3.37 ± 0.61	—	—
Placental weight (kg)	0.72 ± 0.22	—	—
Amniotic fluid volume (mL)	567.74 ± 186.88	—	—
Intraoperative fluid volume (mL)	1211.29 ± 242.80	1191.72 ± 335.18	0.80
Operative time (min)	64.16 ± 12.78	66.72 ± 10.08	0.39

Data are shown as mean (SD).

## Data Availability

The data used to support the findings of this study are included within the article and supplementary information files.
